# Internet-Based Motivation Program for Women With Eating Disorders: Eating Disorder Pathology and Depressive Mood Predict Dropout

**DOI:** 10.2196/jmir.3104

**Published:** 2014-03-31

**Authors:** Ruth von Brachel, Katrin Hötzel, Gerrit Hirschfeld, Elizabeth Rieger, Ulrike Schmidt, Joachim Kosfelder, Tanja Hechler, Dietmar Schulte, Silja Vocks

**Affiliations:** ^1^Department of Clinical Psychology and PsychotherapyOsnabrück UniversityOsnabrückGermany; ^2^Department of Clinical Psychology and PsychotherapyRuhr-University BochumBochumGermany; ^3^Department of Clinical Child and Adolescent PsychologyRuhr-University BochumBochumGermany; ^4^German Paediatric Pain CentreChildren’s Hospital DattelnDattelnGermany; ^5^Research School of PsychologyAustralian National UniversityCanberraAustralia; ^6^Eating Disorders UnitInstitute of Psychiatry at the Maudsley, King’s CollegeUniversity of LondonLondonUnited Kingdom; ^7^Department of Social Sciences and Cultural StudiesUniversity of Applied SciencesDüsseldorfGermany

**Keywords:** attrition, Web-based treatment, eHealth, eating disorders, motivation to change, anorexia nervosa, bulimia nervosa, bootstrapping, survival analysis

## Abstract

**Background:**

One of the main problems of Internet-delivered interventions for a range of disorders is the high dropout rate, yet little is known about the factors associated with this. We recently developed and tested a Web-based 6-session program to enhance motivation to change for women with anorexia nervosa, bulimia nervosa, or related subthreshold eating pathology.

**Objective:**

The aim of the present study was to identify predictors of dropout from this Web program.

**Methods:**

A total of 179 women took part in the study. We used survival analyses (Cox regression) to investigate the predictive effect of eating disorder pathology (assessed by the Eating Disorders Examination-Questionnaire; EDE-Q), depressive mood (Hopkins Symptom Checklist), motivation to change (University of Rhode Island Change Assessment Scale; URICA), and participants’ age at dropout. To identify predictors, we used the least absolute shrinkage and selection operator (LASSO) method.

**Results:**

The dropout rate was 50.8% (91/179) and was equally distributed across the 6 treatment sessions. The LASSO analysis revealed that higher scores on the Shape Concerns subscale of the EDE-Q, a higher frequency of binge eating episodes and vomiting, as well as higher depression scores significantly increased the probability of dropout. However, we did not find any effect of the URICA or age on dropout.

**Conclusions:**

Women with more severe eating disorder pathology and depressive mood had a higher likelihood of dropping out from a Web-based motivational enhancement program. Interventions such as ours need to address the specific needs of women with more severe eating disorder pathology and depressive mood and offer them additional support to prevent them from prematurely discontinuing treatment.

## Introduction

Internet-based interventions have proven to be effective treatments for a variety of psychological disorders [1]. These interventions provide easy access to help for individuals who would otherwise delay seeking treatment and, hence, risk developing a more chronic course [2,3]. The advantages of Web-based interventions seem to be especially valuable in individuals with eating disorders, who are known to seek face-to-face treatment relatively late [4] because of feelings of shame and fear of stigma [5]. Therefore, Web-based interventions for individuals with eating disorders [6-10], their caregivers [11], and individuals at risk for eating disorders [12] have been developed. These interventions yield moderate to large treatment effects [6-14]. Whereas most of these studies investigated Web-based programs for women with bulimic symptoms, online interventions for patients with anorexia nervosa are rare [15].

However, the limitation of such accessible interventions is that they allow participants to easily terminate (ie, with a mouse click) online treatment prematurely [16]. Accordingly, dropout rates for online interventions can be high, ranging from 3% to 81% across trials [17]. The high variance in dropout rates seems to be at least partially because of differences in the intensity and nature of the personal contact offered [18-20]. For example, patients who receive telephone contact instead of only email contact with the therapist have a higher likelihood of staying in online treatment [21].

Beyond the generally elevated dropout rates of Internet interventions, disorder-specific differences also need to be considered. Specifically, individuals with eating disorders may be at particular risk of dropping out from online treatments given their elevated dropout rates from face-to-face treatments [22,23]. Dropout from face-to-face interventions for eating disorders generally ranges from 20% to 40% [22,23]. Research conducted in these settings has identified a number of predictors of treatment dropout for eating disorders. Several studies indicated that patients with higher levels of psychopathology (ie, low self-esteem [24], more severe eating disorder symptoms [25-27], and binge eating/purging subtype of anorexia nervosa [26-28]) at the beginning of treatment are more likely to terminate treatment prematurely. The results concerning comorbid depressive symptoms are mixed. Although the majority of studies have found a higher risk of dropout for patients with higher depression scores [29,30], this effect did not reach significance in a meta-analysis [23]. Additionally, age has been identified as a predictor of staying in treatment in bulimia nervosa because older patients tend to persist longer with face-to-face treatment than younger patients [23]. However, 2 recent meta-analyses found that psychopathology, symptom severity, and age are not stable predictors of dropout from face-to-face therapies for women with eating disorders [22,23].

Another potential predictor of treatment dropout is motivation to change (for a review see [28]) because it has been shown that participants with initially low levels of motivation to change their eating disorder symptoms [31,32] or low cooperativeness scores [27] are more likely to drop out of treatment. Therefore, interventions to enhance motivation to change in women with eating disorders have been implemented and have been shown to reduce dropout as well as enhance motivation to change and reduce eating pathology in some studies [33].

In spite of the growing interest in Internet-delivered interventions for women with eating disorders, there is a dearth of research in this regard, with only 8 studies having investigated predictors of treatment dropout from Internet programs aimed at women with anorexia nervosa, bulimia nervosa, or subthreshold eating disorders [6,8,15,34-38]. Most studies explored dropout by comparing demographic variables and symptom severity in dropouts (women who terminated treatment prematurely) and completers (women who completed treatment). Such a group comparison approach neglects information about the time course of attrition; that is, it does not allow the comparison of different attrition curves according to possible predictors of dropout. Instead, survival analysis seems to be much more suitable for describing and testing whether and when participants drop out [39]. An exception to this can be found in a study by Fernández-Aranda and colleagues [35] who used survival analysis to predict dropout from an Internet-based program for women with bulimia nervosa. The authors found that higher anxiety and lower reward dependence, lower hyperactivity, and lower minimum body mass index (BMI) were predictive of dropout from Internet-delivered cognitive behavioral therapy for bulimia nervosa.

In summary, most studies reporting on dropout from Web-based programs for eating disorders did not find a significant impact of participants’ age on dropout [6,38], although one study found that older patients were less likely to drop out [15]. Symptom severity at the beginning of treatment, including eating disorder pathology [15] and body dissatisfaction [6], has been shown to be positively related to dropout in some studies. However, other researchers found no differences between completers and dropouts concerning eating disorder symptoms [34,38]. A higher level of general psychopathology has been found to increase the likelihood of premature termination of Internet treatment in some studies [6,35]. One study found an effect of depressive and anxious symptoms on dropout [6], whereas 2 other studies found no such effect [37,38].

Taken together, it seems that demographic variables are not reliable predictors of treatment dropout, either in face-to-face or in online settings. In contrast, symptom severity may be a more robust predictor of online treatments, but it has yielded inconclusive results in face-to-face settings [22,23]. Depression has been demonstrated to be a predictor in face-to-face settings, but not in Web-based interventions. Motivation to change is an important predictor of dropout in face-to-face settings, but it has not been studied in online interventions to date.

The aim of the present study was to bridge this gap and to investigate factors leading to dropout from an anonymous Internet-delivered program to enhance motivation to change in individuals with eating disorders [13]. We investigated age, depressive mood, symptom severity, and motivation to change as predictors of dropout from this program. Given the ease of access to and termination of this program, we expected the dropout rate to be relatively high. Based on the studies described previously, we expected participants with higher depression scores, more severe eating pathology, and lower motivation to change to be more likely to terminate treatment early. We did not expect to find any effect of participants’ age. We used survival analysis (ie, Cox regression) to test possible predictors of dropout from the program. Because the number of participants who dropped out was high and, accordingly, the number of events per variable was relatively low, we used the least absolute shrinkage and selection operator (LASSO) method to identify predictors.

## Methods

### Participants

Participants were recruited between March 2011 and March 2012 through newspaper, magazine, and radio announcements as well as via social networks and reports on websites for people with eating disorders. Potential participants completed a self-report screening battery before treatment. The inclusion criteria included female gender and at least one of the following eating disorder symptoms once or more per week within the past 4 weeks (assessed with the Short Evaluation of Eating Disorders [40]): purging, dieting, or excessive exercise. Additionally, a self-reported BMI greater than 15 kg/m^2^ and less than 30 kg/m^2^ was required. Participants who indicated no weight control behaviors at least once per week within the past 4 weeks and participants who reported binge eating only with the absence of any compensatory behaviors were excluded. Furthermore, those with severe depression (a score of 35 or more on the Center for Epidemiologic Studies Depression Scale, CES-D [41]), risk of suicide (assessed with items designed by the authors [13]), severe self-harming behavior (assessed with items designed by the authors [13]), psychotic disorders (a score of 13 or more on the Dutch Screening Device for Psychotic Disorder [42]), dissociative symptoms (a score greater than 8 on the Somatoform Dissociation Questionnaire [43]), substance abuse (a score of 10 or more on the Alcohol Use Disorders Identification Test [44] or the Drug Use Disorders Identification Test [45]), or in current psychotherapy treatment were excluded from the study (for more information on the inclusion and exclusion criteria, see [13]). The final sample consisted of 179 participants who commenced the online program (ie, completed the baseline assessment and received an invitation for the first session).

### Intervention

The online program ESS-KIMO (“Klärendes Internetprogramm zur Steigerung der Veränderungsmotivation bei Essstörungen” or “Internet program to enhance motivation to change in eating disorders”) aims to enhance motivation to change in women with symptoms of anorexia nervosa or bulimia nervosa. The methods and results of the ESS-KIMO have been published elsewhere [13]; this is a secondary analysis of the program. It is based on the transtheoretical model of behavior change [46] and uses the principles of motivational interviewing [47]. It contains 6 weekly online sessions comprising evidence-based interventions to enhance motivation to change [48,49], which are often used in conjunction with motivational interviewing. All aspects of the program and the study took place online and participants remained completely anonymous apart from providing an email address at which they could be contacted. The ESS-KIMO was designed based on the results of previous research [1]. It consisted of individual sessions, a closed website with screening for inclusion and exclusion criteria, and individualized feedback (from RvB or KH) for the required writing tasks in each session. In this feedback, the authors used methods and principles of motivational interviewing, such as selectively reflecting participants’ change or confidence talk and trying to enhance participants’ perception of discrepancies between their current (eating-disordered) behavior and their long-term values and goals.

In general, all sessions dealt with the participants’ ambivalence regarding behavior change. The session content included (1) the typical reasons for and against change in women with eating disorders (eg, participants were asked to make a list of pros and cons); (2) physical and psychological consequences of eating disorders and the eating disorder’s congruity or incongruity with life goals; (3) the eating disorder’s impact on the person’s daily life; (4) self-esteem and its link to the eating disorder; (5) a summary of the aspects that were considered important by the participant regarding behavior change; and (6) information regarding treatment. The content of each session (eg, information given) and the therapeutic tasks were standardized and were the same for each participant. All participants received an invitation to the next session via email 1 week after completing the previous session. During this time, an author (RvB or KH) wrote individualized feedback for the participant’s answer to the writing task. If participants did not log into the program during the 2 days following their invitation, a reminder email was sent on the third day. Up to 3 reminders were sent, each after 3 days. The effectiveness of the program was evaluated in a randomized controlled trial, in which the ESS-KIMO showed promising results for the completers of the program in terms of an improvement in motivation to change and eating disorder symptomology as well as an enhancement of self-esteem [13].

Ethical approval for the study was obtained by the German Psychological Society (Deutsche Gesellschaft für Psychologie, DGPs). Individuals interested in participating received information about the study and were informed that they could withdraw from the study at any time. Participants also received an email address so that they could contact one of the authors (RvB or KH) if they needed additional support. Participants were also given the telephone numbers of emergency contacts in Germany, such as the crisis helpline.

### Measures

#### Overview

In addition to reporting their age, participants completed measures regarding psychopathology and motivation to change at baseline and posttreatment.

#### Eating Disorder Pathology: Eating Disorder Examination-Questionnaire

Eating disorder pathology was assessed with the German version of the Eating Disorder Examination-Questionnaire (EDE-Q) [50,51]. The EDE-Q entails 22 items asking about eating disorder symptoms occurring within the last 28 days, with responses ranging from 0=none to 6=every day. It consists of 4 subscales: Dietary Restraint, Eating Concerns, Weight Concerns, and Shape Concerns. Furthermore, the EDE-Q asks about eating disorder core behaviors (eg, binge eating and purging) and their frequency during the past 28 days. The EDE-Q also allows the calculation of the patient’s self-reported BMI. Similar to the original English-language questionnaire, the German version shows very good internal consistency (Cronbach alpha=.85-.93) and a 3-month test-retest reliability between *r*_tt_=.68 and *r*_tt_=.74 [51,52].

#### Motivation to Change: University of Rhode Island Change Assessment Scale

The University of Rhode Island Change Assessment Scale (URICA) [53] assesses participants’ global motivation to change. It consists of 4 subscales representing the precontemplation, contemplation, action, and maintenance stage of change according to the transtheoretical model [46]. Items are rated on a 5-point Likert scale ranging from 1=disagree strongly to 5=agree strongly. Reliability in women with eating disorders is satisfactory, with internal consistencies between alpha=.58 and alpha=.95 and test-retest reliabilities between *r*_tt_=.58 and *r*_tt_=.73 [54].

#### Depressive Mood: Depression Subscale of the Hopkins Symptom Checklist-25

Depressive mood was assessed with the depression subscale of the German version of the Hopkins Symptom Checklist (HSCL-25) [55-57]. Participants were asked to indicate how strongly they experienced typical depressive symptoms within the past month. The scale has a 4-point Likert format ranging from 1=not at all to 5=very. Reliability in inpatient and outpatient samples is good, with an internal consistency of alpha=.91 and a test-retest reliability of *r*_tt_=.79 to *r*_tt_=.90.

Participants also gave information on a range of different baseline measures including previous treatment, housing situation, marital status, educational level, and experience with the Internet and computers (all assessed with the Biographical Information Questionnaire [58]). They also filled in an assessment of ambivalence (the German Pros and Cons of Eating Disorders Scale [59]), a symptom-specific motivation questionnaire for eating disorders (Stages of Change Questionnaire for Eating Disorders [60]), the Rosenberg Self-Esteem Scale [61], and the Self-Efficacy Scale [62]. These questionnaires were hypothesized as outcome variables in the primary analysis of the ESS-KIMO trial. For the analysis of this study, we based our selection of variables on the literature concerning dropout from face-to-face therapies and Internet programs for women with eating disorders as well as on the restrictions because of the sample size.

### Definition of Dropout

Several authors have pointed out the importance of clearly defining dropout [16,22]. Because we were interested in the predictors of treatment adherence in women with eating disorders, we only investigated dropout from treatment (*treatment dropout*) and not from the study (*study dropout*). That is, we only included participants who had completed all baseline measurements before treatment. Each dropout was patient-initiated; we did not recommend withdrawal from the program at any time. We defined dropout for a particular participant in a particular session when this participant completed the prior session but did not finish the current session during a 4-week time period. We then deleted the participant’s account because of our data privacy protection policy.

### Data Analysis

The present study aimed to test 14 variables as potential predictors of dropping out of the program. These were the EDE-Q scales Dietary Restraint, Eating Concerns, Weight Concerns, and Shape Concerns; participants’ BMI; the frequency of the core eating disorder behaviors binge eating, purging, and excessive exercise; the 4 URICA scales precontemplation, contemplation, action, and maintenance; depressed mood assessed with the HSCL-25; and participants’ age. As recommended [39], we used survival analysis (ie, Cox regression) to test the predictors. Because 91 participants dropped out, the number of events per predictor variable (EPV) was much lower than the recommended 10 EPV [63]. Therefore, we used the LASSO to identify relevant predictors [64] because this method yields reliable estimates in such scenarios [65]. Because this method is not invariant to linear scaling, we standardized the individual variables before the analysis. The variability of the LASSO estimates is typically assessed using a simple bootstrapping approach [66]. Specifically, we drew (with replacement) 1000 pseudosamples from our full sample and calculated the LASSO. For each pseudosample, the optimal lambda was calculated by using a crossvalidation procedure with 50 folds. The smallest lambda was then chosen to calculate the LASSO estimates, and the resulting estimates were recorded. Descriptive statistics (mean, SD, 95% CI) were used to inspect these estimates. The software R was used for data analysis [67].

## Results

### Baseline Characteristics

The core demographic characteristics of participants at the beginning of the program are shown in Table 1. The maximum BMI reported (30.47 kg/m^2^) is based on participants’ responses to the EDE-Q directly before the beginning of the intervention, whereas eligibility for the study (ie, a maximum BMI of 30) was determined based on participants’ earlier responses to the screening battery. Some participants differed in their answers between the screening and preassessment time points given the time lag between these assessments (eg, participants in the waiting-list condition waited 8 weeks until the beginning of the program).

**Table 1:** Participants’ characteristics at baseline.

Variable		Mean (SD)	Minimum	Maximum
Age		27.54 (8.02)	18.00	59.00
Body mass index (kg/m^2^)		20.56 (3.49)	15.06	30.47
**Eating Disorder Examination-Questionnaire (EDE-Q)**				
	Eating Concerns	2.93 (1.30)	0.20	5.60
	Dietary Restraint	3.76 (1.35)	0.20	6.00
	Shape Concerns	4.17 (1.29)	0.50	6.00
	Weight Concerns	3.75 (1.37)	0.40	6.00
	Binge eating episodes (past 28 days)	9.91 (8.74)	0.00	28.00
	Vomiting frequency (past 28 days)	8.43 (10.28)	0.00	28.00
	Excessive exercise	6.28 (8.14)	0.00	28.00
**University of Rhode Island Change Assessment Scale (URICA)**				
	Precontemplation	1.61 (0.57)	1.00	4.50
	Contemplation	4.20 (0.53)	1.25	5.00
	Action	3.74 (0.60)	1.50	5.00
	Maintenance	3.35 (0.76)	1.25	5.00
**Hopkins Symptom Checklist (HSCL-25)**				
	Depression	2.40 (0.63)	1.17	3.83

### Overall Dropout

**Figure 1:**
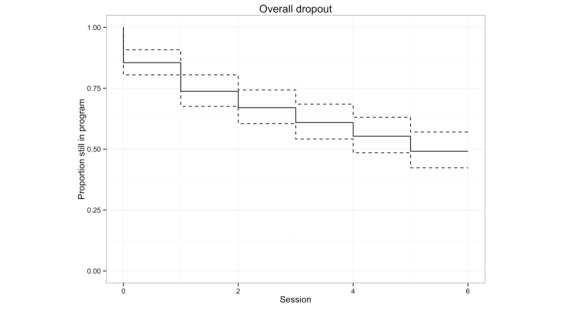
Overall dropout of participants during the course of the program (broken lines represent 95% CI).

Of the 179 participants who completed the first session, 91 (50.8%) dropped out at some point during the program. On average, the participants completed mean 4.92 (SD 2.4) out of the 6 sessions. The dropout was equally distributed across the sessions (Figure 1), with approximately 10% of the participants dropping out after each session.

### Predictors of Dropout

**Figure 2:**
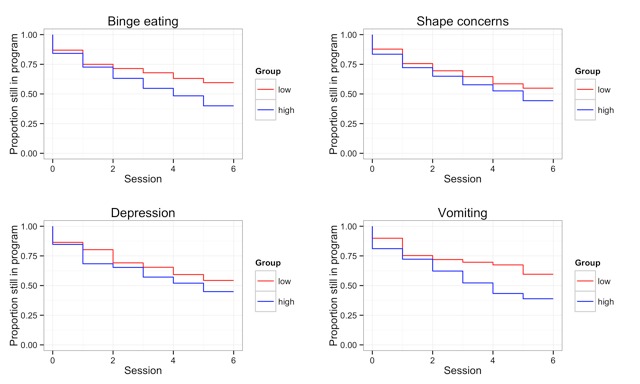
Dropout stratified by high vs low levels of the relevant variables based on median split.

As shown in Table 2, the LASSO method identified the EDE-Q subscale Shape Concerns, frequency of vomiting within the past 28 days, frequency of binge eating within the past 28 days, and the HSCL-25 depression score with Cox regression as relevant predictors of dropout within the full sample. Results indicated that participants with high levels of Shape Concerns and depression as well as frequent binge eating episodes and vomiting were more likely to drop out of the program (see Figure 2). All other variables, including age, the URICA scores, BMI, and the EDE-Q scales Restraint, Eating Concerns, and Weight Concerns were not significantly related to dropout.

**Table 2:** Model parameters.

Variable	Full sample	Bootstrapping results		
	LASSO estimate	Mean (SD)	95% CI	*P*
EDE-Q Shape Concerns	.07	.07 (.10)	.06, .08	<.001
Vomiting	.03	.07 (.09)	.06, .07	<.001
HSCL-25 depression	.05	.06 (.08)	.05, .06	<.001
Binge eating	.14	.10 (.09)	.09, .10	<.001

### Bootstrapping LASSO

**Figure 3:**
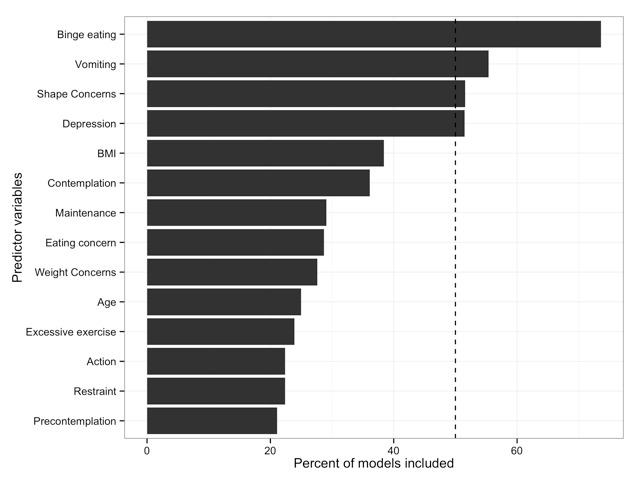
Number of pseudosamples for which the LASSO identified the variables as relevant.

By using bootstrapping to estimate the variability of these results, we found that the 4 variables identified as predictors in the full sample were also the only variables that were identified in more than 50% of the bootstrapping samples (see Figure 3). Across samples, the frequency of binge eating, the frequency of vomiting, the EDE-Q subscale Shape Concern, and depressive mood were identified as predictors of participants remaining in the program. Importantly, the bootstrapped confidence intervals of these estimates did not include zero, indicating that these are larger than expected by chance effects alone.

## Discussion

### Principal Findings

The primary aim of the present study was to identify predictors of dropping out of an online program for women with eating disorders. We found that 50.8% (91/179) of the participants terminated their participation in the program prematurely. Our analysis identified 4 indexes of symptom severity that predicted dropout, namely the EDE-Q Shape Concerns subscale, frequency of vomiting within the previous 28 days, frequency of binge eating within the previous 28 days, and the HSCL-25 depression subscale. Neither motivation to change nor age predicted dropout.

The overall level of symptom severity in the present sample was high when comparing the scores of the present sample with the published norms of the EDE-Q [51]. This shows that a large proportion of participants suffered from significant eating disorder pathology, although diagnoses were not made by means of structured clinical interviews. Therefore, the sample seems to be suitable for drawing assumptions about women with eating disorders in general.

Similarly, the overall level of the dropout rate is comparable to that from other Web-based programs in general [17]. The current program was completely anonymous, which might have made it easier for participants to prematurely terminate their participation. Future research should evaluate the importance of anonymity in Web-based interventions for individuals with eating disorders and determine its role in dropout.

The dropout rate was equally distributed across the 6 sessions. However, it remains unclear whether the reasons for early versus late dropout are identical [22,39]. It is possible that early dropout is primarily because of participants’ dissatisfaction with the program, whereas late dropout may also be because of progress withdrawal (ie, participants feeling that they have already benefited enough) [22,39,68]. Speculative reasons for program withdrawal in the study may have included feeling dissatisfied with the motivational content and the absence of practical advice and strategies for overcoming the eating disorder, feeling overwhelmed by negative emotions when dealing with one’s own reasons for and against the eating disorder, or having resolved motivational issues faster than in 6 sessions [22]. Because dropout was distributed evenly across the 6 sessions, the specific content of any particular session can be discarded as a cause of dropout.

Although the high dropout rate is fairly typical for Web-based interventions [19], a high dropout rate is related to poorer outcome [16], meaning that it is important to understand the factors predicting early termination. Concerning the predictors of dropout, various indexes of symptom severity (ie, the EDE-Q Shape Concerns subscale as well as the frequency of binge eating and vomiting) predicted premature discontinuation of the Internet program. The finding regarding Shape Concerns is consistent with results from research in women with eating disorders in face-to-face settings [25,26] as well as previous Web-based interventions [6], which also showed that higher body dissatisfaction makes treatment dropout more likely. The finding that the higher the frequency of binge eating and vomiting, the more likely participants were to terminate treatment early is also in-line with previous findings. Accordingly, Kahn and Pike [26] and Woodside and colleagues [28] found that patients with the binge eating/purging type of anorexia nervosa were also more likely to drop out of face-to-face treatments. The commonly high levels of impulsivity in this patient group may also facilitate treatment dropout [28]. Because higher symptom severity is associated with longer duration of illness, it may reflect that these patients are also more reluctant to confront their maladaptive behavior [25]. The relationship between higher eating disorder pathology and premature termination of the online treatment program observed in the present study suggests that the online modality may be insufficient for those with more severe eating disorders.

We also found that participants with higher levels of depressive mood were more likely to drop out of our program. This finding is in accordance with previous findings from face-to-face settings for eating disorders [29,30] and a study on a Web-based treatment program for women with eating disorders [6]. However, other studies have reported a lack of association between depression and dropout from Web-based programs for women with eating disorders [37,38]. It seems plausible that an impact of depression which emerges in conventional therapies could also be relevant in Web-based treatment because the contents of the Web-based treatments were the same as the contents of face-to face therapies (ie, mostly consisting of cognitive behavioral therapy and motivational interviewing; eg, [5,36]). Participants in the present study showed high depression scores, whereas the participants of other Web-based interventions for eating disorders reported only moderate depressive symptoms [37,38]. Thus, it is possible that the effect of depression on treatment dropout only emerges for participants with relatively severe depressive symptomatology, for whom programs such as ours may be inadequate because they fail to target depressive features [13]. It is important to note that although we excluded participants with severely elevated depressive symptoms, our cutoff point of 35 on the CES-D is still relatively high [69], meaning that participants with elevated depressive symptoms were included in the study. Moreover, the fact that depressive mood was predictive of treatment termination although participants with extreme scores were a priori excluded from the study underscores that even moderate levels of depression may constitute a contraindication to engaging in such programs.

It is also noteworthy that several of the tested variables were not significant predictors of treatment dropout. In accordance with previous studies [6,38], we did not find any effect of age on dropout although the age range in our study was relatively wide, which should have facilitated the detection of any existing age effect. Furthermore, we did not find any effect of motivation to change as assessed by the URICA on treatment retention, despite the fact that motivation to change seems to be a stable predictor in face-to-face therapies [31,32,70]. Given that our program dealt exclusively with participants’ motivation to change and aimed specifically at enhancing motivation, it may have been helpful to negate the impact of low motivation on treatment adherence. It is plausible that motivation to change is a more robust predictor of treatment adherence in interventions without a motivational focus, such as conventional cognitive behavioral therapy [71]. In contrast, in most studies, motivation to change was not investigated as a predictor of treatment retention in face-to-face programs aimed at enhancing motivation to change [72,73]. An exception to this is the study by Allen and colleagues [74] who directly investigated the role of motivation to change in predicting treatment dropout from a motivational program. They did not find any effect on most of the measures, with the exception that higher scores on the precontemplation scale were predictive of dropout. To sum up, motivation to change seems to be a more robust predictor of treatment retention in interventions targeting change rather than motivation to change. This may be because motivational programs help to increase participants’ motivation; hence, eliminating the adverse effects of low motivation on dropout. Furthermore, the limited variance in motivation to change in this study may have made it more difficult to detect any effect of motivation. The limited variance in the URICA scores in our study is comparable to those from other studies on motivational enhancement programs [75,76], with the highest scores on the contemplation scale. It is perhaps not surprising that participants were primarily ambivalent about change given that participants would have needed a minimum amount of motivation to register for the program and they believed that they could benefit from a motivational program.

### Limitations

Several limitations should be kept in mind when interpreting the results of the present study. The complete anonymity and lack of direct contact made it impossible to obtain clinical diagnoses. Although the high scores on the EDE-Q indicate a clinical level of symptom severity, because we were unable to distinguish between people with different eating disorder diagnoses, we were also unable to test for diagnosis as a predictor of dropout. Furthermore, we used a global measure of motivation to change, which has been criticized in the context of eating disorders [77] because a patient’s motivation to change her eating disorder may vary between different symptoms. For example, motivation to change binge eating is usually higher than motivation to change dietary restriction [78]. Therefore, some authors argue that it is not adequate to assess a patient’s motivation to change globally for the eating disorders as a whole [79]. It is possible that we were unable to detect an effect of motivation to change because of the global assessment of the construct.

Nevertheless, this is the first study investigating a wide range of predictors of dropout from a Web-based program using adequate methodology in a large sample of women suffering from a wide range of eating disorder pathology.

### Conclusions

This study identifies symptom severity and depressive symptoms as predictors of dropout from a Web-based program targeting motivation to change in women with symptoms of an eating disorder. In contrast, retention in the program was not dependent on age or initial motivation. These findings have the potential to guide further development of Web-based treatment programs in terms of tailoring online-delivered therapies to the specific needs of patients. For example, women who indicate high levels of depression could be offered an additional module that addresses affective disturbance. Furthermore, women who suffer from more severe eating disorder pathology might need more therapeutic guidance or additional contact via telephone or in a face-to-face setting to help them to continue the program. Although high dropout rates remain problematic in online treatments, there is still a large proportion of patients (particularly those with less severe eating disorders and depressive symptoms) who complete this easily accessible means of delivering therapeutic interventions.

## Abbreviations

BMI: body mass index
CES-D: Center for Epidemiologic Studies Depression Scale
EDE-Q: Eating Disorders Examination-Questionnaire
EPV: events per predictor variable
HSCL-25: Hopkins Symptom Checklist
LASSO: least absolute shrinkage and selection operator
URICA: University of Rhode Island Change Assessment Scale
